# Analysis of Markov Jump Processes under Terminal Constraints

**DOI:** 10.1007/978-3-030-72016-2_12

**Published:** 2021-03-01

**Authors:** Michael Backenköhler, Luca Bortolussi, Gerrit Großmann, Verena Wolf

**Affiliations:** 8 Eindhoven University of Technology, Eindhoven, The Netherlands; 9 Aalborg University, Aalborg East, Denmark; 10 Saarbrücken Graduate School of Computer Science, Saarland University, Saarland Informatics Campus E1 3, Saarbrücken, Germany; 11 Univeristy of Trieste, Trieste, Italy; 12 Saarland University, Saarland Informatics Campus E1 3, Saarbrücken, Germany

**Keywords:** Bayesian Inference, Bridging problem, Smoothing, Lumping, Rare Events.

## Abstract

Many probabilistic inference problems such as stochastic filtering or the computation of rare event probabilities require model analysis under initial and terminal constraints. We propose a solution to this *bridging problem* for the widely used class of population-structured Markov jump processes. The method is based on a state-space lumping scheme that aggregates states in a grid structure. The resulting approximate bridging distribution is used to iteratively refine relevant and truncate irrelevant parts of the state-space. This way, the algorithm learns a well-justified finite-state projection yielding guaranteed lower bounds for the system behavior under endpoint constraints. We demonstrate the method’s applicability to a wide range of problems such as Bayesian inference and the analysis of rare events.

### 1 Introduction

Discrete-valued continuous-time Markov Jump Processes (MJP) are widely used to model the time evolution of complex discrete phenomena in continuous time. Such problems naturally occur in a wide range of areas such as chemistry [16], systems biology [49,46], epidemiology [36] as well as queuing systems [10] and finance [39]. In many applications, an MJP describes the stochastic interaction of populations of agents. The state variables are counts of individual entities of different populations.

Many tasks, such as the analysis of rare events or the inference of agent counts under partial observations naturally introduce terminal constraints on the system. In these cases, the system's initial state is known, as well as the system's (partial) state at a later time-point. The probabilities corresponding to this so-called *bridging problem* are often referred to as *bridging probabilities* [17,19]. For instance, if the exact, full state of the process $X_t$ has been observed at time 0 and $T$, the bridging distribution is given by

$$\Pr(X_t = x \mid X_0 = x_0, X_T = x_g)$$

for all states $x$ and times $t \in [0, T]$. Often, the condition is more complex, such that in addition to an initial distribution, a terminal distribution is present. Such problems typically arise in a Bayesian setting, where the a priori behavior of a system is filtered such that the posterior behavior is compatible with noisy, partial observations [11,25]. For example, time-series data of protein levels is available while the mRNA concentration is not [1,25]. In such a scenario our method can be used to identify a good truncation to analyze the probabilities of mRNA levels.

Bridging probabilities also appear in the context of rare events. Here, the rare event is the terminal constraint because we are only interested in paths containing the event. Typically researchers have to resort to Monte-carlo simulations in combination with variance reduction techniques in such cases [14,26].

Efficient numerical approaches that are not based on sampling or ad-hoc approximations have rarely been developed.

Here, we combine state-of-the-art truncation strategies based on a forward analysis [28,4] with a refinement approach that starts from an abstract MJP with lumped states. We base this lumping on a grid-like partitioning of the state-space. Throughout a lumped state, we assume a uniform distribution that gives an efficient and convenient abstraction of the original MJP. Note that the lumping does not follow the classical paradigm of Markov chain lumpability [12] or its variants [15]. Instead of an approximate block structure of the transition-matrix used in that context, we base our partitioning on a segmentation of the molecule counts. Moreover, during the iterative refinement of our abstraction, we identify those regions of the state-space that contribute most to the bridging distribution. In particular, we refine those lumped states that have a bridging probability above a certain threshold $\delta$ and truncate all other macro-states. This way, the algorithm learns a truncation capturing most of the bridging probabilities. This truncation provides guaranteed lower bounds because it is at the granularity of the original model.

In the rest of the paper, after presenting related work (Section 2) and background (Section 3), we discuss the method (Section 4) and several applications, including the computation of rare event probabilities as well as Bayesian smoothing and filtering (Section 5).

### 2 Related Work

The problem of endpoint constrained analysis occurs in the context of Bayesian estimation [41]. For population-structured MJPs, this problem has been addressed by Huang et al. [25] using moment closure approximations and by Wildner and Köppl [48] further employing variational inference. Golightly and Sherlock modified stochastic simulation algorithms to approximatively augment generated trajectories [17]. Since a statistically exact augmentation is only possible for few simple cases, diffusion approximations [18] and moment approximations [35] have been employed. Such approximations, however, do not give any guarantees on the approximation error and may suffer from numerical instabilities [43].

The bridging problem also arises during the estimation of first passage times and rare event analysis. Approaches for first-passage times are often of heuristic nature [42,22,8]. Rigorous approaches yielding guaranteed bounds are currently limited by the performance of state-of-the-art optimization software [6]. In biological applications, rare events of interest are typically related to the reachability of certain thresholds on molecule counts or mode switching [45]. Most methods for the estimation of rare event probabilities rely on importance sampling [26,14]. For other queries, alternative variance reduction techniques such as control variates are available [5]. Apart from sampling-based approaches, dynamic finite-state projections have been employed by Mikeev et al. [34], but are lacking automated truncation schemes.

The analysis of countably infinite state-spaces is often handled by a predefined truncation [27]. Sophisticated state-space truncations for the (unconditioned) forward analysis have been developed to give lower bounds and rely on a trade-off between computational load and tightness of the bound [37,28,4,24,31].

Reachability analysis, which is relevant in the context of probabilistic verification [8,38], is a bridging problem where the endpoint constraint is the visit of a set of goal states. Backward probabilities are commonly used to compute reachability likelihoods [2,50]. Approximate techniques for reachability, based on moment closure and stochastic approximation, have also been developed in [8,9], but lack error guarantees. There is also a conceptual similarity between computing bridging probabilities and the forward-backward algorithm for computing state-wise posterior marginals in hidden Markov models (HMMs) [40]. Like MJPs, HMMs are a generative model that can be conditioned on observations. We only consider two observations (initial and terminal state) that are not necessarily noisy but the forward and backward probabilities admit the same meaning.

## 3 Preliminaries

### 3.1 Markov Jump Processes with Population Structure

A population-structured Markov jump process (MJP) describes the stochastic interactions among agents of distinct types in a well-stirred reactor. The assumption of all agents being equally distributed in space, allows to only keep track of the overall copy number of agents for each type. Therefore the state-space is $\mathcal{S} \subseteq \mathbb{N}^{n_S}$ where $n_S$ denotes the number of agent types or populations. Interactions between agents are expressed as *reactions*. These reactions have associated gains and losses of agents, given by non-negative integer vectors $v_j^-$ and $v_j^+$ for reaction $j$, respectively. The overall effect is given by $v_j = v_j^+ - v_j^-$. A reaction between agents of types $S_1, \dots, S_{n_S}$ is specified in the following form:

$$\sum_{\ell=1}^{n_S} v_{j\ell}^- S_\ell \xrightarrow{\alpha_j(x)} \sum_{\ell=1}^{n_S} v_{j\ell}^+ S_\ell \,. \tag{1}$$

The propensity function $\alpha_j$ gives the rate of the exponentially distributed firing time of the reaction as a function of the current system state $x \in \mathcal{S}$. In population models, *mass-action* propensities are most common. In this case the firing rate is given by the product of the number of reactant combinations in $x$ and a *rate constant* $c_j$, i.e.

$$\alpha_j(x) := c_j \prod_{\ell=1}^{n_S} \binom{x_\ell}{v_{j\ell}^-} . \tag{2}$$

In this case, we give the rate constant in (1) instead of the function $\alpha_j$. For a given set of $n_R$ reactions, we define a stochastic process $\{X_t\}_{t\geq 0}$ describing the evolution of the population sizes over time $t$. Due to the assumption of exponentially distributed firing times, $X$ is a continuous-time Markov chain (CTMC) on $\mathcal{S}$ with infinitesimal generator matrix $Q$, where the entries of $Q$ are

$$Q_{x,y} = \begin{cases} \sum_{j:x+v_j=y} \alpha_j(x) , & \text{if } x \neq y, \\ -\sum_{j=1}^{n_R} \alpha_j(x) , & \text{otherwise.} \end{cases} \tag{3}$$

The probability distribution over time can be analyzed as an initial value problem. Given an initial state $x_0$, the distribution[1]

$$\pi(x_i, t) = \Pr(X_t = x_i \mid X_0 = x_0), \quad t \geq 0 \tag{4}$$

evolves according to the Kolmogorov forward equation

$$\frac{d}{dt}\pi(t) = \pi(t)Q , \tag{5}$$

where $\pi(t)$ is an arbitrary vectorization $(\pi(x_1, t), \pi(x_2, t), \ldots, \pi(x_{|\mathcal{S}|}, t))$ of the states.

Let $x_g \in \mathcal{S}$ be a fixed goal state. Given the terminal constraint $\Pr(X_T = x_g)$ for some $T \geq 0$, we are interested in the so-called backward probabilities

$$\beta(x_i, t) = \Pr(X_T = x_g \mid X_t = x_i), \quad t \leq T . \tag{6}$$

Note that $\beta(\cdot, t)$ is a function of the conditional event and thus is no probability distribution over the state-space. Instead $\beta(\cdot, t)$ gives the reaching probabilities for all states over the time span of $[t, T]$. To compute these probabilities, we can employ the Kolmogorov backward equation

$$\frac{d}{dt}\beta(t) = Q\beta(t)^\top , \tag{7}$$

where we use the same vectorization to construct $\beta(t)$ as we used for $\pi(t)$. The above equation is integrated backwards in time and yields the reachability probability for each state $x_i$ and time $t < T$ of ending up in $x_g$ at time $T$.

[1] In the sequel, $x_i$ denotes a state with index $i$ instead of its $i$-th component.

The state-space of many MJPs with population structure, even simple ones, is countably infinite. In this case, we have to truncate the state-space to a *reasonable* finite subset. The choice of this truncation heavily depends on the goal of the analysis. If one is interested in the most "common" behavior, for example, a dynamic mass-based truncation scheme is most appropriate [32]. Such a scheme truncates states with small probability during the numerical integration. However, common mass-based truncation schemes are not as useful for the bridging problem. This is because trajectories that meet the specific terminal constraints can be far off the main bulk of the probability mass. We solve this problem by a state-space lumping in connection with an iterative refinement scheme.

Consider as an example a birth-death process. This model can be used to model a wide variety of phenomena and often constitutes a sub-module of larger models. For example, it can be interpreted as an M/M/1 queue with service rates being linearly dependent on the queue length. Note, that even for this simple model, the state-space is countably infinite.

**Model 1 (Birth-Death Process).** *The model consists of exponentially distributed arrivals and service times proportional to queue length. It can be expressed using two mass-action reactions:*

$$\varnothing \xrightarrow{10} X \qquad \text{and} \qquad X \xrightarrow{.1} \varnothing\,.$$

*The initial condition $X_0 = 0$ holds with probability one.*

#### 3.2 Bridging Distribution

The process' probability distribution given both initial and terminal constraints is formally described by the conditional probabilities

$$\gamma(x_i, t) = \Pr(X_t = x_i \mid X_0 = x_0, X_T = x_g), \quad 0 \le t \le T \tag{8}$$

for fixed initial state $x_0$ and terminal state $x_g$. We call these probabilities the *bridging probabilities*. It is straight-forward to see that $\gamma$ admits the factorization

$$\gamma(x_i, t) = \pi(x_i, t)\beta(x_i, t)/\pi(x_g, T) \tag{9}$$

due to the Markov property. The normalization factor, given by the reachability probability $\pi(x_g, T) = \beta(x_0, 0)$, ensures that $\gamma(\cdot, t)$ is a distribution for all time points $t \in [0, T]$. We call each $\gamma(\cdot, t)$ a *bridging distribution*. From the Kolmogorov equations (5) and (7) we can obtain both the forward probabilities $\pi(\cdot, t)$ and the backward probabilities $\beta(\cdot, t)$ for $t < T$.

We can easily extend this procedure to deal with hitting times constrained by a finite time-horizon by making the goal state $x_g$ absorbing.

In Figure 1 we plot the forward, backward, and bridging probabilities for Model 1. The probabilities are computed on a $[0, 100]$ state-space truncation. The approximate forward solution $\hat{\pi}$ shows how the probability mass drifts upwards towards the stationary distribution Poisson(100). The backward probabilities

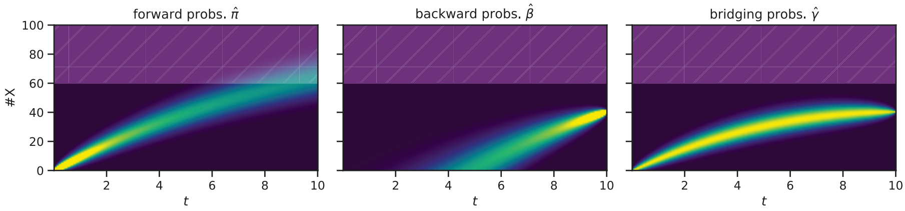

**Fig. 1.** Forward, backward, and bridging probabilities for Model 1 with initial constraint $X_0 = 0$ and terminal constraint $X_{10} = 40$ on a truncated state-space. Probabilities over 0.1 in $\hat{\pi}$ and $\hat{\beta}$ are given full intensity for visual clarity. The lightly shaded area ($\geq 60$) indicates a region being more relevant for the forward than for the bridging probabilities.

are highest for states below the goal state $x_g = 40$. This is expected because upwards drift makes reaching $x_g$ more probable for "lower" states. Finally, the approximate bridging distribution $\hat{\gamma}$ can be recognized to be proportional to the product of forward $\hat{\pi}$ and backward probabilities $\hat{\beta}$.

### 4 Bridge Truncation via Lumping Approximations

We first discuss the truncation of countably infinite state-spaces to analyze backward and forward probabilities (Section 4.1). To identify effective truncations we employ a lumping scheme. In Section 4.2, we explain the construction of macro-states and assumptions made, as well as the efficient calculation of transition rates between them. Finally, in Section 4.3 we present an iterative refinement algorithm yielding a suitable truncation for the bridging problem.

#### 4.1 Finite State Projection

Even in simple models such as a birth-death Process (Model 1), the reachable state-space is countably infinite. Direct analyzes of backward (6) and forward equations (4) are often infeasible. Instead, the integration of these differential equations requires working with a finite subset of the infinite state-space [37]. If states are truncated, their incoming transitions from states that are not truncated can be re-directed to a *sink state*. The accumulated probability in this sink state is then used as an error estimate for the forward integration scheme. Consequently, many truncation schemes, such as dynamic truncations [4], aim to minimize the amount of "lost mass" of the forward probability. We use the same truncation method but base the truncation on bridging probabilities rather than the forward probabilities.

#### 4.2 State-Space Lumping

When dealing with bridging problems, the most likely trajectories from the initial to the terminal state are typically not known a priori. Especially if the event in question is rare, obtaining a state-space truncation adapted to its constraints is difficult. We devise a lumping scheme that groups nearby states, i.e. molecule counts, into larger *macro-states*. A macro-state is a collection of states treated as one state in a lumped model, which can be seen as an abstraction of the original model. These macro-states form a partitioning of the state-space. In this lumped model, we assume a uniform distribution over the constituent micro-states inside each macro-state. Thus, given that the system is in a particular macro-state, all of its micro-states are equally likely. This partitioning allows us to analyze significant regions of the state-space efficiently albeit under a rough approximation of the dynamics. Iterative refinement of the state-space after each analysis moves the dynamics closer to the original model. In the final step of the iteration, the considered system states are at the granularity of the original model such that no approximation error is introduced by assumptions of the lumping scheme. Computational efficiency is retained by truncating in each iteration step those states that contribute little probability mass to the (approximated) bridging distributions.

We choose a lumping scheme based on a grid of hypercube macro-states whose endpoints belong to a predefined grid. This topology makes the computation of transition rates between macro-states particularly convenient. Mass-action reaction rates, for example, can be given in a closed-form due to the Faulhaber formulae. More complicated rate functions such as Hill functions can often be handled as well by taking appropriate integrals.

Our choice is a scheme that uses $n_S$-dimensional hypercubes. A macro-state $\bar{x}_i(\ell^{(i)}, u^{(i)})$ (denoted by $\bar{x}_i$ for notational ease) can therefore be described by two vectors $\ell^{(i)}$ and $u^{(i)}$. The vector $\ell^{(i)}$ gives the corner closest to the origin, while $u^{(i)}$ gives the corner farthest from the origin. Formally,

$$\bar{x}_i = \bar{x}_i(\ell^{(i)}, u^{(i)}) = \{x \in \mathbb{N}^{n_S} \mid \ell^{(i)} \leq x \leq u^{(i)}\}, \tag{10}$$

where '$\leq$' stands for the element-wise comparison. This choice of topology makes the computation of transition rates between macro-states particularly convenient: Suppose we are interested in the set of micro-states in macro-state $\bar{x}_i$ that can transition to macro-state $\bar{x}_k$ via reaction $j$. It is easy to see that this set is itself an interval-defined macro-state $\bar{x}_{i \xrightarrow{j} k}$. To compute this macro-state we can simply shift $\bar{x}_i$ by $v_j$, take the intersection with $\bar{x}_k$ and project this set back. Formally,

$$\bar{x}_{i \xrightarrow{j} k} = ((\bar{x}_i + v_j) \cap \bar{x}_k) - v_j \,, \tag{11}$$

where the additions are applied element-wise to all states making up the macro-states. For the correct handling of the truncation it is useful to define a general exit state

$$\bar{x}_{i \xrightarrow{j}} = ((\bar{x}_i + v_j) \setminus \bar{x}_i) - v_j. \tag{12}$$

This state captures all micro-states inside $\bar{x}_i$ that can leave the state via reaction $j$. Note that all operations preserve the structure of a macro-state as defined in (10). Since a macro-state is based on intervals the computation of the transition rate is often straight-forward. Under the assumption of polynomial rates, as

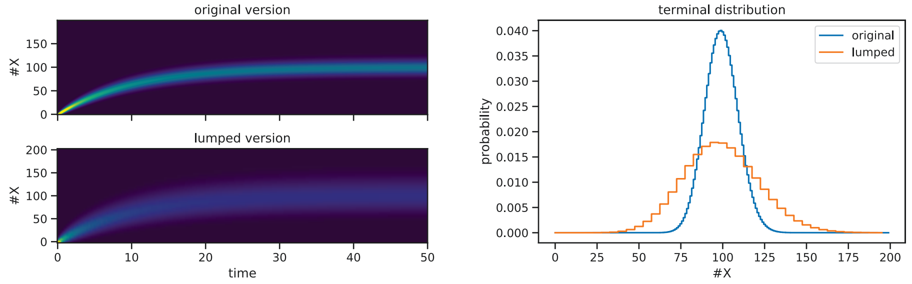

**Fig. 2.** A lumping approximation of Model 1 on the state-space truncation to $[0, 200]$ on $t \in [0, 50]$. On the left-hand side solutions of a regular truncation approximation and a lumped truncation (macro-state size is 5) are given. On the right-hand side the respective terminal distributions $\Pr(X_{50} = x_i)$ are contrasted.

it is the case for mass-action systems, we can compute the sum of rates over this transition set efficiently using Faulhaber's formula. We define the lumped transition function

$$\bar{\alpha}_j(\bar{x}) = \sum_{x \in \bar{x}} \alpha_j(x) \tag{13}$$

for macro-state $\bar{x}$ and reaction $j$. As an example consider the following mass-action reaction $2X \xrightarrow{c} \varnothing$. For macro-state $\bar{x} = \{0, \ldots, n\}$ we can compute the corresponding lumped transition rate

$$\bar{\alpha}(\bar{x}) = \frac{c}{2} \sum_{i=1}^{n} i(i-1) = \frac{c}{2} \sum_{i=1}^{n} (i^2 - i) = \frac{c}{2} \left( \frac{2n^3 + 3n^2 + n}{6} - \frac{n^2 + n}{2} \right)$$

eliminating the explicit summation in the lumped propensity function.

For polynomial propensity functions $\alpha$ such formulae are easily obtained automatically. For non-polynomial propensity functions, we can use the continuous integral as an approximation. This is demonstrated on a case study in Section 5.2.

Using the transition set computation (11) and the lumped propensity function (13) we can populate the $Q$-matrix of the finite lumping approximation:

$$\bar{Q}_{\bar{x}_i, \bar{x}_k} = \begin{cases} \sum_{j=1}^{n_R} \bar{\alpha}_j \left( \bar{x}_{i \xrightarrow{j} k} \right) / \text{vol}\left(\bar{x}_i\right), & \text{if } \bar{x}_i \neq \bar{x}_k \\ -\sum_{j=1}^{n_R} \bar{\alpha}_j \left( \bar{x}_{i \xrightarrow{j}} \right) / \text{vol}\left(\bar{x}_i\right), & \text{otherwise} \end{cases} \tag{14}$$

In addition to the lumped rate function over the transition state $\bar{x}_{i \xrightarrow{j} k}$, we need to divide by the total volume of the lumped state $\bar{x}_i$. This is due to the assumption of a uniform distribution inside the macro-states. Using this $Q$-matrix, we can compute the forward and backward solution using the respective Kolmogorov equations (5) and (7).

Interestingly, the lumped distribution tends to be less concentrated. This is due to the assumption of a uniform distribution inside macro-states. This effect is illustrated by the example of a birth-death process in Figure 2. Due to this effect, an iterative refinement typically keeps an over-approximation in terms of state-space area. This is a desirable feature since relevant regions are less likely to be pruned due to lumping approximations.

#### 4.3 Iterative Refinement Algorithm

The iterative refinement algorithm (Alg. 1) starts with a set of large macro-states that are iteratively refined, based on approximate solutions to the bridging problem. We start by constructing square macro-states of size $2^m$ in each dimension for some $m \in \mathbb{N}$ such that they form a large-scale grid $\mathcal{S}^{(0)}$. Hence, each initial macro-state has a volume of $(2^m)^{n_S}$. This choice of grid size is convenient because we can halve states in each dimension. Moreover, this choice ensures that all states have equal volume and we end up with states of volume $2^0 = 1$ which is equivalent to a truncation of the original non-lumped state-space.

An iteration of the state-space refinement starts by computing both the forward and backward probabilities (lines 2 and 3) via integration of (5) and (7), respectively, using the lumped $\hat{Q}$-matrix. Based on the resulting approximate forward and backward probabilities, we compute an approximation of the bridging distributions (line 4). This is done for each time-point in an equispaced grid on $[0, T]$. The time grid granularity is a hyper-parameter of the algorithm. If the grid is too fine, the memory overhead of storing backward $\hat{\beta}^{(i)}$ and forward solutions $\hat{\pi}^{(i)}$ increases.[2] If, on the other hand, the granularity is too low, too much of the state-space might be truncated. Based on a threshold parameter $\delta > 0$ states are either removed or split (line 7), depending on the mass assigned to them by the approximate bridging probabilities $\hat{\gamma}_t^{(i)}$. A state can be split by the `split`-function which halves the state in each dimension. Otherwise, it is removed. Thus, each macro-state is either split into $2^{n_S}$ new states or removed entirely. The result forms the next lumped state-space $\mathcal{S}^{(i+1)}$. The $Q$-matrix is adjusted (line 10) such that transition rates for $\mathcal{S}^{(i+1)}$ are calculated according to (14). Entries of truncated states are removed from the transition matrix. Transitions leading to them are re-directed to a sink state (see Section 4.1). After $m$ iterations (we started with states of side lengths $2^m$) we have a standard finite state projection scheme on the original model tailored to computing an approximation of the bridging distribution.

In Figure 3 we give a demonstration of how Algorithm 1 works to refine the state-space iteratively. Starting with an initial lumped state-space $\mathcal{S}^{(0)}$ covering a large area of the state-space, repeated evaluations of the bridging distributions are performed. After five iterations the remaining truncation includes all states that significantly contribute to the bridging probabilities over the times $[0, T]$.

It is important to realize that determining the most relevant states is *the* main challenge. The above algorithm solves this problem by considering only

[2] We denote the approximations with a hat (e.g. $\hat{\pi}$) rather than a bar (e.g. $\bar{\pi}$) to indicate that not only the lumping approximation but also a truncation is applied and similarly for the $Q$-matrix.

**Algorithm 1:** Iterative refinement for the bridging problem

**input** : Initial partitioning $\mathcal{S}^{(0)}$, truncation threshold $\delta$
**output:** approximate bridging distribution $\hat{\gamma}$

1 **for** $i = 1, \ldots, m$ **do**
2 $\quad \hat{\pi}_t^{(i)} \leftarrow$ solve approximate forward equation on $\mathcal{S}^{(i)}$;
3 $\quad \hat{\beta}_t^{(i)} \leftarrow$ solve approximate backward equation on $\mathcal{S}^{(i)}$;
4 $\quad \hat{\gamma}_t^{(i)} \leftarrow \hat{\beta}^{(i)} \hat{\pi}^{(i)} / \hat{\pi}(x_g, T)$; `/* approximate bridging distribution */`
5 $\quad \mathcal{S}^{(i+1)} \leftarrow \emptyset$;
6 $\quad$ **foreach** $\bar{x} \in \mathcal{S}^{(i)}$ **do**
7 $\quad\quad$ **if** $\exists t. \hat{\gamma}_t^{(i)}(\bar{x}) \geq \delta$; `/* refine based on bridging probabilities */`
8 $\quad\quad$ **then**
9 $\quad\quad\quad \mathcal{S}^{(i+1)} \leftarrow \mathcal{S}^{(i+1)} \cup$ `split`$(\bar{x})$;
10 $\quad$ update $\hat{Q}$-matrix;
11 **return** $\hat{\gamma}^{(i)}$;

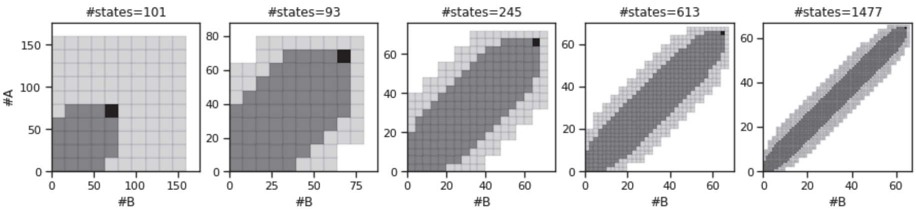

**Fig. 3.** The state-space refinement algorithm on two parallel unit-rate arrival processes. The bridging problem from $(0, 0)$ to $(64, 64)$ and $T = 10$ and truncation threshold $\delta = 5\text{e-}3$. States with a bridging probability below $\delta$ are light grey. The macro-state containing the goal state is marked in black. The initial macro-states are of size $16 \times 16$.

those parts of the state-space that contribute most to the bridging probabilities. The truncation is tailored to this condition and might ignore regions that are likely in the unconditioned case. For instance, in Fig. 1 the bridging probabilities mostly remain below a population threshold of $\#X = 60$ (as indicated by the lighter/darker coloring), while the forward probabilities mostly exceed this bound. Hence, in this example a significant portion of the forward probabilities $\hat{\pi}_t^{(i)}$ is captured by the sink state. However, the condition in line 7 in Algorithm 1 ensures that states contributing significantly to $\hat{\gamma}_t^{(i)}$ will be kept and refined in the next iteration.

### 5 Results

We present four examples in this section to evaluate our proposed method. A prototype was implemented in Python 3.8. For numerical integration we

| threshold $\delta$ | 1e-2 | 1e-3 | 1e-4 | 1e-5 |
|---|---|---|---|---|
| truncation size | 1154 | 2354 | 3170 | 3898 |
| overall states | 2074 | 3546 | 4586 | 5450 |
| estimate | 8.8851e-30 | 1.8557e-29 | 1.8625e-29 | 1.8625e-29 |
| rel. error | 5.2297e-01 | 3.6667e-03 | 3.7423e-05 | 9.5259e-08 |

**Table 1.** Estimated reachability probabilities based on varying truncation thresholds $\delta$: The true probability is 1.8625e-29. We also report the size of the final truncation and the accumulated size of all truncations during refinement iterations (overall states).

used the Scipy implementation [47] of the implicit method based on backward-differentiation formulas [13]. The analysis as a Jupyter notebook is made available online[3].

#### 5.1 Bounding Rare Event Probabilities

We consider a simple model of two parallel Poisson processes describing the production of two types of agents. The corresponding probability distribution has Poisson product form at all time points $t \geq 0$ and hence we can compare the accuracy of our numerical results with the exact analytic solution. We use the proposed approach to compute lower bounds for rare event probabilities. [4]

**Model 2 (Parallel Poisson Processes).** *The model consists of two parallel independent Poisson processes with unit rates.*

$$\varnothing \xrightarrow{1} A \qquad \text{and} \qquad \varnothing \xrightarrow{1} B\,.$$

*The initial condition $X_0 = (0, 0)$ holds with probability one. After $t$ time units each species abundance is Poisson distributed with rate $\lambda = t$.*

We consider the final constraint of reaching a state where both processes exceed a threshold of 64 at time 20. Without prior knowledge, a reasonable truncation would have been $160 \times 160$. But our analysis shows that just 20% of the states are necessary to capture over 99.6% of the probability mass reaching the target event (cf. Table 1). Decreasing the threshold $\delta$ leads to a larger set of states retained after truncation as more of the bridging distribution is included (cf. Figure 4). We observe an increase in truncation size that is approximately logarithmic in $\delta$, which, in this example, indicates robustness of the method with respect to the choice of $\delta$.

[3] https://www.github.com/mbackenkoehler/mjp_bridging

[4] These bounds are rigorous up to the approximation error of the numerical integration scheme. However, the forward solution could be replaced by an adaptive uniformization approach [3] for a more rigorous integration error control.

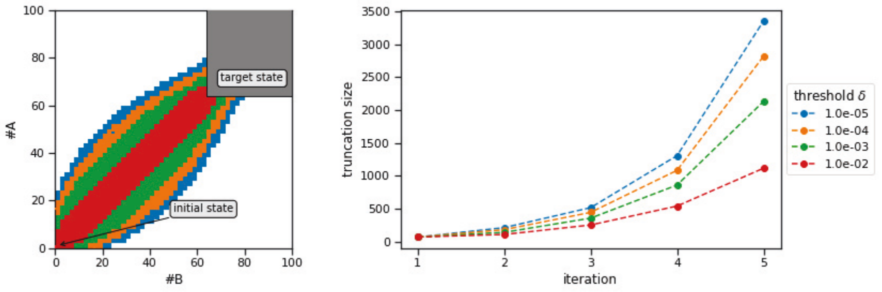

**Fig. 4.** State-space truncation for varying values of the threshold parameter $\delta$: Two parallel Poisson processes under terminal constraints $X_{20}^{(A)} \geq 64$ and $X_{20}^{(B)} \geq 64$. The initial macro-states are $16 \times 16$ such that the final states are regular micro states.

*Comparison to other methods* The truncation approach that we apply is similar to the one used by Mikeev et al. [34] for rare event estimation. However, they used a given linearly biased MJP model to obtain a truncation. A general strategy to compute an appropriate biasing was not proposed. It is possible to adapt our truncation approach to the dynamic scheme in Ref. [34] where states are removed in an on-the-fly fashion during numerical integration.

A finite state-space truncation covering the same area as the initial lumping approximation would contain 25,600 states.[5] The standard approach would be to build up the entire state-space for such a model [27]. Even using a conservative truncation threshold $\delta$ = 1e-5, our method yields an accurate estimate using only about a fifth (5450) of this accumulated over all intermediate lumped approximations.

### 5.2 Mode Switching

Mode switching occurs in models exhibiting *multi-modal* behavior [44] when a trajectory traverses a potential barrier from one mode to another. Often, mode switching is a rare event and occurs in the context of gene regulatory networks where a mode is characterized by the set of genes being currently active [30]. Similar dynamics also commonly occur in queuing models where a system may for example switch its operating behavior stochastically if traffic increases above or decreases below certain thresholds. Using the presented method, we can get both a qualitative and quantitative understanding of switching behavior without resorting to Monte-Carlo methods such as importance sampling.

**Exclusive Switch** The exclusive switch [7] has three different modes of operation, depending on the DNA state, i.e. on whether a protein of type one or two is bound to the DNA.

[5] Here, the goal is not treated as a single state. Otherwise, it consists of 24,130 states.

**Model 3 (Exclusive Switch).** *The exclusive switch model consists of a promoter region that can express both proteins $P_1$ and $P_2$. Both can bind to the region, suppressing the expression of the other protein. For certain parameterizations, this leads to a bi-modal or even tri-modal behavior.*

$$D \xrightarrow{\rho} D + P_1 \qquad D \xrightarrow{\rho} D + P_2 \qquad P_1 \xrightarrow{\lambda} \varnothing \qquad P_2 \xrightarrow{\lambda} \varnothing$$

$$D + P_1 \xrightarrow{\beta} D.P_1 \qquad D.P1 \xrightarrow{\gamma} D + P_1 \qquad D.P_1 \xrightarrow{\alpha} D.P_1 + P_1$$

$$D + P_2 \xrightarrow{\beta} D.P_2 \qquad D.P2 \xrightarrow{\gamma} D + P_2 \qquad D.P_2 \xrightarrow{\alpha} D.P_2 + P_2$$

*The parameter values are $\rho =$ 1e-1, $\lambda =$ 1e-3, $\beta =$ 1e-2, $\gamma =$ 8e-3, and $\alpha =$ 1e-1.*

Since we know a priori of the three distinct operating modes, we adjust the method slightly: The state-space for the DNA states is not lumped. Instead we "stack" lumped approximations of the $P_1$-$P_2$ phase space upon each other. Special treatment of DNA states is common for such models [28].

To analyze the switching, we choose the transition from (variable order: $P_1$, $P_2$, $D$, $D.P_1$, $D.P_2$) $x_1 = (32, 0, 0, 0, 1)$ to $x_2 = (0, 32, 0, 1, 0)$ over the time interval $t \in [0, 10]$. The initial lumping scheme covers up to 80 molecules of $P_1$ and $P_2$ for each mode. Macro-states have size $8 \times 8$ and the truncation threshold is $\delta =$ 1e-4.

In the analysis of biological switches, not only the switching probability but also the switching dynamics is a central part of understanding the underlying biological mechanisms. In Figure 5 (left), we therefore plot the time-varying probabilities of the gene state conditioned on the mode. We observe a rapid unbinding of $P_2$, followed by a slow increase of the binding probability for $P_1$. These dynamics are already qualitatively captured by the first lumped approximation (dashed lines).

**Toggle Switch** Next, we apply our method to a toggle switch model exhibiting non-polynomial rate functions. This well-known model considers two proteins $A$ and $B$ inhibiting the production of the respective other protein [29].

**Model 4.** *Toggle Switch (Hill functions) We have population types $A$ and $B$ with the following reactions and reaction rates.*

$$\varnothing \xrightarrow{\alpha_1(\cdot)} A\,, \quad \textit{where} \quad \alpha_1(x) = \frac{\rho}{1 + x_B}, \qquad A \xrightarrow{\lambda} \varnothing$$

$$\varnothing \xrightarrow{\alpha_1(\cdot)} B\,, \quad \textit{where} \quad \alpha_1(x) = \frac{\rho}{1 + x_A}, \qquad B \xrightarrow{\lambda} \varnothing$$

*The parameterization is $\rho = 10$, $\lambda = 0.1$.*

Due to the non-polynomial rate functions $\alpha_1$ and $\alpha_2$, the transition rates between macro-states are approximated by using the continuous integral

$$\bar{\alpha}_1(\bar{x}) \approx \int_{a-0.5}^{b+0.5} \frac{\rho}{1 + x}\, dx = \rho \left( \log \left( b + 1.5 \right) - \log \left( a + 0.5 \right) \right)$$

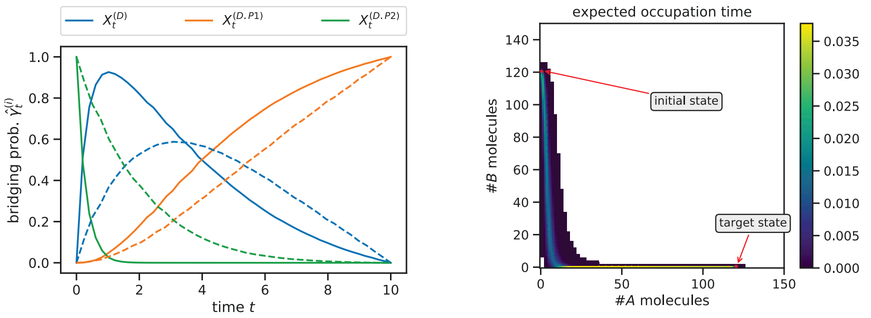

**Fig. 5.** (left) Mode probabilities of the exclusive switch bridging problem over time for the first lumped approximation (dashed lines) and the final approximation (solid lines) with constraints $X_0 = (32, 0, 0, 1, 0)$ and $X_{10} = (0, 32, 0, 0, 1)$. (right) The expected occupation time (excluding initial and terminal states) for the switching problem of the toggle switch using Hill-type functions. The bridging problem is from initial $(0, 120)$ to a first passage of $(120, 0)$ in $t \in [0, 10]$.

for a macro-state $\bar{x} = \{a, \ldots, b\}$.

We analyze the switching scenario from $(0, 120)$ to the first visit of state $(120, 0)$ up to time $T = 10$. The initial lumping scheme covers up to 352 molecules of $A$ and $B$ and macro-states have size $32 \times 32$. The truncation threshold is $\delta =$ 1e-4. The resulting truncation is shown in Figure 5 (right). It also illustrates the kind of insights that can be obtained from the bridging distributions. For an overview of the switching dynamics, we look at the expected occupation time under the terminal constraint of having entered state $(120, 0)$. Letting the corresponding hitting time be $\tau = \inf\{t \geq 0 \mid X_t = (120, 0)\}$, the expected occupation time for some state $x$ is $E\left(\int_0^\tau 1_{=x}(X_t)\, dt \mid \tau \leq 10\right)$. We observe that in this example the switching behavior seems to be asymmetrical. The main mass seems to pass through an area where initially a small number of $A$ molecules is produced followed by a total decay of $B$ molecules.

#### 5.3 Recursive Bayesian Estimation

We now turn to the method's application in recursive Bayesian estimation. This is the problem of estimating the system's past, present, and future behavior under given observations. Thus, the MJP becomes a hidden Markov model (HMM). The observations in such models are usually noisy, meaning that we cannot infer the system state with certainty.

This estimation problem entails more general distributional constraints on terminal $\beta(\cdot, T)$ and initial $\pi(\cdot, 0)$ distributions than the point mass distributions considered up until now. We can easily extend the forward and backward probabilities to more general initial distributions and terminal distributions $\beta(T)$. For the forward probabilities we get

$$\pi(x_i, t) = \sum_j \Pr(X_t = x_i \mid X_0 = x_j)\pi(x_j, 0), \tag{15}$$

and similarly the backward probabilities are given by

$$\beta(x_i, t) = \sum_j \Pr(X_T = x_j \mid X_t = x_i)\beta_T(x_j)\,. \tag{16}$$

We apply our method to an SEIR (susceptible-exposed-infected-removed) model. This is widely used to describe the spreading of an epidemic such as the current COVID-19 outbreak [23,20]. Temporal snapshots of the epidemic spread are mostly only available for a subset of the population and suffer from inaccuracies of diagnostic tests. Bayesian estimation can then be used to infer the spreading dynamics given uncertain temporal snapshots.

**Model 5 (Epidemics Model).** *A population of susceptible individuals can contract a disease from infected agents. In this case, they are exposed, meaning they will become infected but cannot yet infect others. After being infected, individuals change to the removed state. The mass-action reactions are as follows.*

$$S + I \xrightarrow{\lambda} E + I \qquad E \xrightarrow{\mu} I \qquad I \xrightarrow{\rho} R$$

*The parameter values are* $\lambda = 0.5$*,* $\mu = 3$*,* $\rho = 3$*. Due to the stoichiometric invariant* $X_t^{(S)} + X_t^{(E)} + X_t^{(I)} + X_t^{(R)} = \text{const.}$*, we can eliminate* $R$ *from the system.*

We consider the following scenario: We know that initially ($t = 0$) one individual is infected and the rest is susceptible. At time $t = 0.3$ all individuals are tested for the disease. The test, however, only identifies infected individuals with probability 0.99. Moreover, the probability of a false positive is 0.05. We like to identify the distribution given both the initial state and the measurement at time $t = 0.3$. In particular, we want to infer the distribution over the latent counts of $S$ and $E$ by *recursive Bayesian estimation.*

The posterior for $n_I$ infected individuals at time $t$, given measurement $Y_t = \hat{n}_I$ can be computed using Bayes' rule

$$\Pr(X_t^{(I)} = n_I \mid Y_t = \hat{n}_I) \propto \Pr(Y_t = \hat{n}_I \mid X_t^{(I)} = n_I) \Pr(X_t^{(I)} = n_I)\,. \tag{17}$$

This problem is an extension of the bridging problem discussed up until now. The difference is that the terminal posterior is estimated it using the result of the lumped forward equation and the measurement distribution using (17). Based on this estimated terminal posterior, we compute the bridging probabilities and refine the truncation tailored to the location of the posterior distribution. In Figure 6 (left), we illustrate the bridging distribution between the terminal posterior and initial distribution. In the context of filtering problems this is commonly referred to as smoothing. Using the learned truncation, we can obtain the posterior distribution for the number of infected individuals at $t = 0.3$ (Figure 6 (middle)). Moreover, can we infer a distribution over the unknown number of susceptible and exposed individuals (Figure 6 (right)).

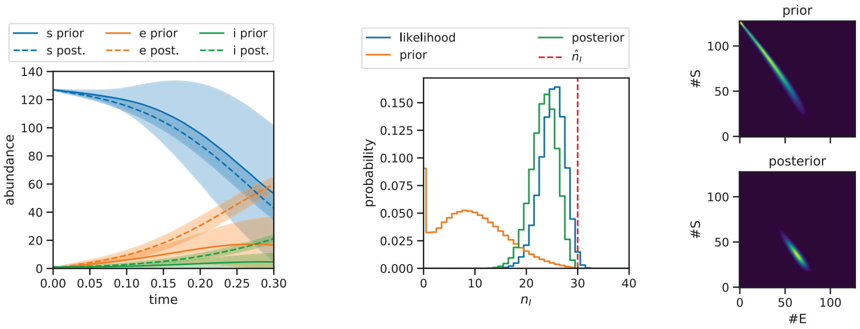

**Fig. 6.** (left) A comparison of the prior dynamics and the posterior smoothing (bridging) dynamics. (middle) The prior, likelihood, and posterior of the number of infected individuals $n_I$ at time $t = 0.3$ given the measurement $\hat{n}_I = 30$. (right) The prior and posterior distribution over the latent types $E$ and $S$.

### 6 Conclusion

The analysis of Markov Jump processes with constraints on the initial and terminal behavior is an important part of many probabilistic inference tasks such as parameter estimation using Bayesian or maximum likelihood estimation, inference of latent system behavior, the estimation of rare event probabilities, and reachability analysis for the verification of temporal properties. If endpoint constraints correspond to atypical system behaviors, standard analysis methods fail as they have no strategy to identify those parts of the state-space relevant for meeting the terminal constraint.

Here, we proposed a method that is not based on stochastic sampling and statistical estimation but provides a direct numerical approach. It starts with an abstract lumped model, which is iteratively refined such that only those parts of the model are considered that contribute to the probabilities of interest. In the final step of the iteration, we operate at the granularity of the original model and compute lower bounds for these bridging probabilities that are rigorous up to the error of the numerical integration scheme.

Our method exploits the population structure of the model, which is present in many important application fields of MJPs. Based on experience with other work based on truncation, the approach can be expected to scale up to at least a few million states [33]. Compared to previous work, our method neither relies on approximations of unknown accuracy nor additional information such as a suitable change of measure in the case of importance sampling. It only requires a truncation threshold and an initial choice for the macro-state sizes.

In future work, we plan to extend our method to hybrid approaches, in which a moment representation is employed for large populations while discrete counts are maintained for small populations. Moreover, we will apply our method to model checking where constraints are described by some temporal logic [21].

**Acknowledgements** This project was supported by the DFG project MULTIMODE and Italian PRIN project SEDUCE.
